# Adequacy of surgical margins, re-excision, and evaluation of factors associated with recurrence: a retrospective study of 769 basal cell carcinomas^☆^

**DOI:** 10.1016/j.abd.2022.07.005

**Published:** 2023-03-16

**Authors:** Yıldız Gürsel Ürün, Nuray Can, Merve Bağış, Sezgi Sarıkaya Solak, Mustafa Ürün

**Affiliations:** a Department of Dermatology, Faculty of Medicine, Trakya University, Edirne, Turkey; b Department of Pathology, Faculty of Medicine, Trakya University, Edirne, Turkey

**Keywords:** Carcinoma, Basal cell, Margins of excision, Neoplasm metastasis, Recurrence, Risk factor

## Abstract

**Background:**

Achieving adequate surgical margins and preventing recurrence are important in the treatment of basal cell carcinoma (BCC).

**Objectives:**

The objectives of this study were to evaluate the adequacy of surgical margins and the re-excision rates in patients with primary BCC who underwent standard surgical treatment using our proposed algorithm and to define the risk factors in patients with recurrent BCC.

**Methods:**

The medical records of patients who were histopathologically diagnosed with BCC were reviewed. An algorithm created based on previous literature was used to determine the distribution of optimal surgical margins adequacy and re-excision rates.

**Results:**

Statistically significant differences were observed between the cases with and without recurrence in age at diagnosis (p = 0.004), tumor size (p = 0.023), tumor location in the H zone of the face (p = 0.005), and aggressive histopathological subtype (p = 0.000). When the tumors were evaluated for adequacy of deep and lateral surgical margins and re-excision rates, higher rates of adequate excision (457 cases, 68.0%) and re-excision (43 cases, 33.9%) were noted for tumors in the H or M zone.

**Study limitations:**

Inadequate follow-up of newly diagnosed patients in terms of recurrence and metastasis and the retrospective application of our proposed algorithm are the limitations of the present study.

**Conclusions:**

Our results showed that if BCC was detected at an early age and at an early stage, recurrence was lower. The H and M zones were the regions with the highest rates of optimal surgical outcomes.

## Introduction

Basal Cell Carcinoma (BCC) is the most common cancer in people with white skin, and its incidence is increasing worldwide. BCCs are a heterogeneous group of tumors with clinical and histopathological features.^1^ Clinically, they present as skin-colored, erythematous, or brownish-reddish nodules, plaques, or ulcers, depending on the site of involvement and stage of the disease.^2^ Depending on the risk of tumor recurrence, the histopathological subtypes of BCCs are classified as follows: Low-risk BCCs: nodular, superficial, infundibulocystic (a variant of BCC with adnexal differentiation), and fibroepithelial; High-risk: micronodular, infiltrating, sclerosing/morphoeic, and basosquamous carcinoma.^3^

Most BCCs occur on the head and neck (i.e., regions exposed to sun), followed by the trunk and extremities (i.e., relatively unexposed).^3^, ^4^ Ultraviolet (UV) exposure is the most important carcinogenic factor.^1^ Other risk factors include male gender, Fitzpatrick types I and II, a personal history of BCC, chronic arsenic exposure, exposure to ionizing radiation, long-term immunosuppression, genetic syndromes, and family history.^4^

In most cases of primary BCC, surgical treatment is recommended.^1^ The primary goal of treatment is to prevent a recurrence.^5^ Risk factors for recurrence include histopathological subtype and presence of perineural invasion, tumor location and size, tumor margins, primary or recurrent tumor, and patient-related factors (immunosuppression, location of prior radiotherapy).^6^ Optimal surgical margins were established to prevent a recurrence. However, incomplete excision in anatomical regions such as the face usually cannot be performed due to functional and cosmetic problems.^7^

The primary objective of this study is to evaluate the adequacy ratios of the surgical margin with our proposed algorithm in selecting the safe margin in patients undergoing standard surgical treatment and to evaluate the distribution ratios of tumors that are re-excised after standard surgical treatment. Our secondary objective is to determine the predictive risk factors that cause recurrence in recurrent BCC cases and to review the clinical features, histopathological findings, and treatment protocols of metastatic BCC cases. In addition, the authors aim to analyze the epidemiological, clinical, and histopathological features of BCC and compare them with previously published reports.

## Methods

### Study design and ethics

In this study, a total of 769 patients who were admitted to our institution between January 1, 2011, and December 30, 2021, and who had a confirmed diagnosis of BCC by histopathology were retrospectively evaluated. For patients with more than one primary BCC, data were analyzed based on the first tumor in the study period. The presence of more than one concurrent lesion in a patient was considered a single case. Approval was obtained from the ethics committee of Trakya University Faculty of Medicine (2021/422).

### Exclusion criteria, clinical and histological data

After reviewing the medical records, patients under 18 years of age, with incomplete data, and with a BCC diagnosis by punch or incisional biopsy were excluded from the study. Medical records were reviewed for demographic information (gender, age at diagnosis, time since diagnosis), clinical features (ulcer, actinic keratosis, and immunosuppression of the lesion), tumor features (tumor location, sun-exposed body site, tumor size, depth of invasion, patients with one or more tumors), and histopathological subtype. Considering the study by Li et al., the accepted sun-exposed regions were the head and neck, forearms, wrists, and lower legs.^8^

In addition, the positivity of deep or lateral surgical margins and treatments applied, postoperative follow-up data, recurrence, and metastasis was recorded. The term “recurrence” was used for clinically detectable tumors during the follow-up period, not only for residual tumors. Regional Metastases (RM) were defined as those that had metastasized to regional lymph nodes, soft tissue (including subcutaneous tissue or skin), salivary glands, or ipsilateral muscle, bone and cartilage in the same anatomic region.^9^ Criteria for the diagnosis of Distant Metastases (DM) in BCC include tumor metastases from a primary cutaneous BCC lesion at distant, noncontiguous sites that have histopathological features similar to those of the primary BCC.^10^

**Figure 1:**
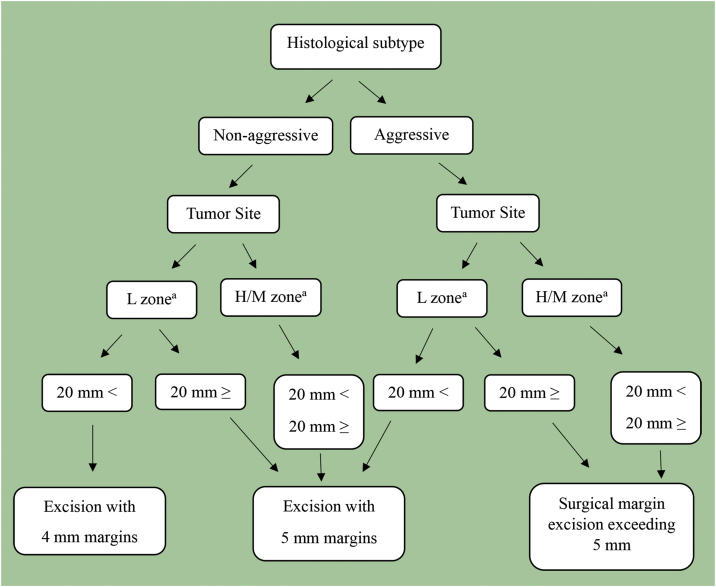
Algorithm for optimal surgical margin of primary basal cell carcinoma. ^a^ H zone; “mask areas” of the face (central face, eyelids, eyebrows, periorbital, nose, lips [cutaneous and vermilion], chin, mandible, preauricular and postauricular skin/sulci, temple, ear), genitalia, hands, and feet. M zone; cheeks, forehead, scalp, neck, and pretibia. L zone; trunk and extremities (excluding pretibia, hands, feet, nail units, and ankles).

According to the WHO classification, histopathological subtypes were divided into two groups: Low-risk and high-risk.^3^ Low-risk BCCs and high-risk BCCs were accepted as nonaggressive and aggressive histopathological subtypes. Therefore, they were used in the proposed algorithm. Patients with two different histopathological subtypes in the same specimen were considered mixed types.^11^ Considering the tumor location, patients were assigned to H zone = “mask areas” of the face (central face, eyelids, eyebrows, periorbital, nose, lips [skin and vermilion], chin, mandible, preauricular and postauricular skin/sulci, temple, ear), genitals, hands, and feet; Zone M = cheeks, forehead, scalp, neck, and pretibia; Zone L = trunk and extremities (except pretibia, hands, feet, nail beds, and ankles). Based on the most recent 2022 revision of the National Comprehensive Cancer Network (NCCN) guideline, those whose tumor was in zones H and M, regardless of tumor size, and those whose tumor was larger than ≥20 mm and was in zone L were classified as high-risk. As seen in Fig. 1, a new algorithm was proposed by utilizing the algorithm created by Luz et al. for primary BCC in 2015.^12^ In contrast to the algorithm of Luz et al., the new NCCM risk recommendations for BCC recurrence were followed in the risk assessment of tumor location and size. When determining the adequacy of surgical margins, those with recurrent and/or metastatic tumors were not included in the evaluation.

In addition, the adequacy of surgical margins and re-excision rates for BCC was also evaluated following the NCCN Basal Cell Skin Cancer guidelines published in 2016.^13^ Tumor characteristics in our proposed algorithm were rearranged in line with the recommendations of this guideline, and the adequacy of surgical margins and re-excision rates were re-evaluated.

### Statistical analysis

Statistical analysis of the data was performed using the program IBM SPSS version 26. Categorical data were summarized as (n) and (%) and continuous variables as median (min‒max). Since continuous variables were not normally distributed (Kolmogorov Smirnov and Shapiro-Wilk p < 0.05), Mann-Whitney *U* statistical analysis was used to compare the two groups. Univariate logistic regression analysis evaluated the factors effective for disease recurrence, and p < 0.05 was considered statistically significant.

## Results

The result of the study was that 455 (59.2%) of the 769 patients were male and 314 (40.8%) were female. The age of the patients ranged from 23 to 99 years (mean 69 years) for BCC. In 614 (79.8%) of the patients, the time since diagnosis of BCC was more than 3 years (Supplementary Table 1).

### Anatomic distribution

**Figure 2:**
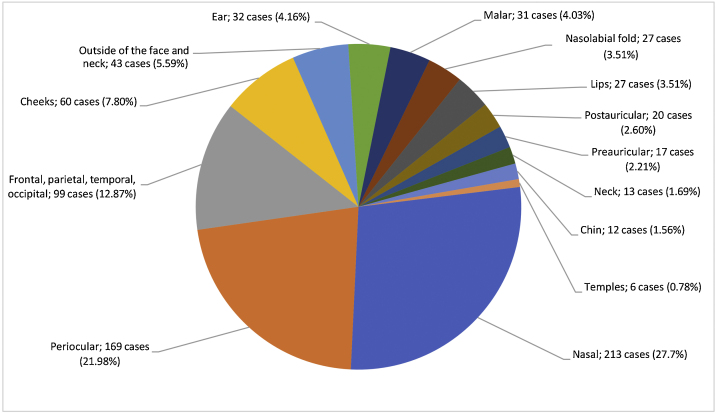
Distribution of basal cell carcinoma by tumor site^a^. (^a^ Periorbital region, eyelids, and eyebrows were evaluated within the periocular region. Nasal region is used instead of nose).

In 726 (94.4%) patients, most tumors were located in the head and neck region. When analyzing tumors in the head and neck, they were most frequently located in the nasal region (213 cases, 27.7%), followed by the periorbital region (169 cases, 21.9%) (Fig. 2).

### Histopathological subtype for BCC

**Figure 3:**
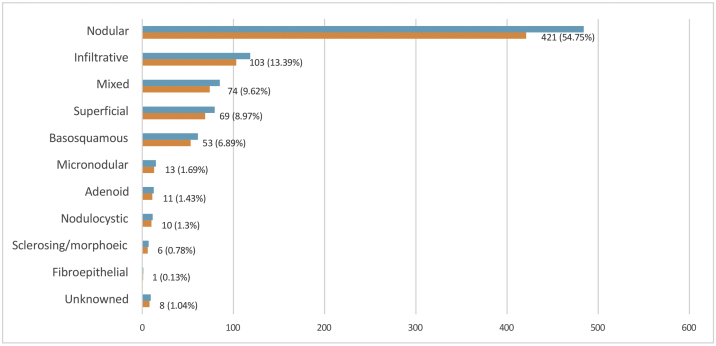
Distribution of basal cell carcinoma by histopatolological subtype, n (%).

Histopathological subtyping was not available for 8 of 769 cases. These cases were categorized as “unknown”. When the remaining 761 BCC patients were evaluated, the most common subtype was a nodular type (421 cases, 56.7%), followed by an infiltrative type (103 cases, 13.3%) (Fig. 3).

### Tumor size and depth of invasion

**Figure 4:**
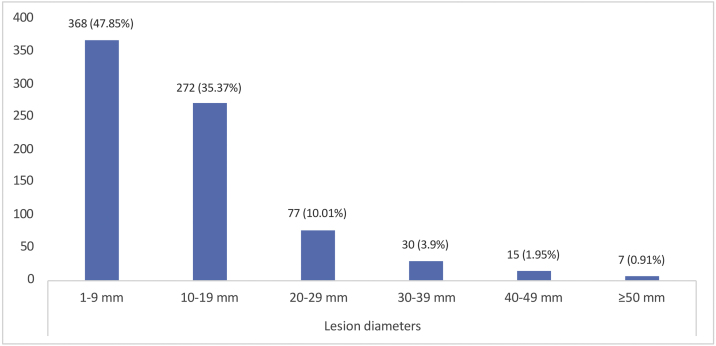
Distribution of maximum lesion diameters for basal cell carcinoma (mm, millimeter).

The mean tumor diameter was 12.09 ± 9.76 mm. When the distribution of tumor diameters was evaluated, 368 cases (47.8%) were between 1‒9 mm. Giant BCC (tumor size ≥50 mm) was detected in 7 patients (0.91%) (Fig. 4).

The mean depth of invasion was 3.92 ± 4.12 mm. Histopathologically, the aggressive subtype was detected in 250 cases (32.9%). When comparing the depth of invasion of nonaggressive and aggressive tumors, there was a statistically significant difference (p = 0.015) (Table 1).

**Table 1:** Depth of invasion of nonaggressive and aggressive tumors (n = 761).

Lesion	Mean (SD)	Median (range)	p	95% CI
Nonaggressive (n = 511, 67.1%)	3.75 (4.06)	3 (0.5‒65)	*0.015*	0.012‒0.019
Aggressive (n = 250, 32.9%)	4.3 (4.28)	3 (0.5‒35)		

SD, Standard Deviation; CI, Confidence Intervals.

Mann Whitney U analysis; p-values < 0.05 were considered to be statistically significant.

**Figure 5:**
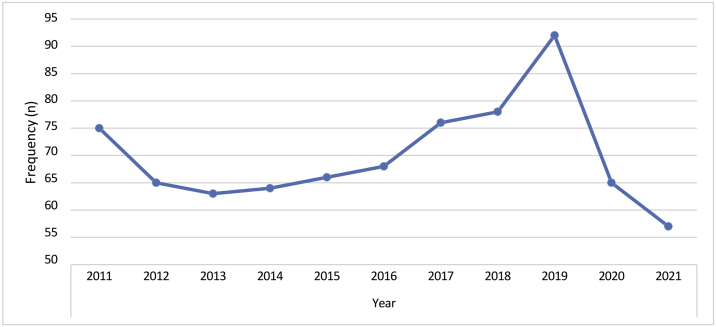
Annual number of cases in our institute with diagnosed basal cell carcinoma between 2011 and 2021 (n = 769).

A mean of 70 BCC cases (9.09%) per year was detected in our department. The decrease in the number of cases in 2020 and 2021 was related to the pandemic COVID-19 (Fig. 5).

### Recurrence

During the 10-year study period, local recurrence occurred in 74 (9.6%) of 769 cases. Time to recurrence averaged 4.51 ± 17.18 months. Risk factors (male gender, age at diagnosis, presence of ulcer in the lesion, immunosuppression, tumor location in the H zone, tumor size greater than 20 mm, histopathologically aggressive subtypes, individuals with multiple BCC at diagnosis) were assessed in patients with recurrence. When comparing these features between patients with and without recurrence, the variables age at diagnosis (p = 0.004), tumor size (p = 0.023), tumor location in the H zone of the face (p = 0.005), and presence of an aggressive subtype (p = 0.005; p = 0.000) showed a statistically significant difference (Table 2).

**Table 2:** Characteristics of tumors as potential risk factors for recurrence.

	Non-Recurrence (%)	Recurrence (%)	p-value	HR (95% CI)
Gender (Male)	413 (59.4)	42 (56.8)	0.657	1.116 (0.688‒1.811)
Age at diagnosis	68.16 (13.5)	72.96 (12.53)	***0.004***	1.029 (1.009‒1.049)
Ulcer	387 (55.7)	50 (67.6)	0.052	1.658 (0.996‒2.759)
Immunosuppression^a^	38 (7)	2 (3.6)	0.348	0.5 (0.117‒2.129)
Tumor size (≥20 mm)^b^	70 (10.1)	14 (18.9)	***0.023***	2.083 (1.107‒3.92)
Tumor location in H zone of the face^b^	489 (70.4)	64 (86.5)	***0.005***	2.696 (1.358‒5.354)
Aggressive^b^	203 (29.5)	47 (63.5)	***0.000***	4.15 (2.515‒6.849)
Patients with multiple BCC at diagnosis	90 (12.9)	14 (18.9)	0.156	1.569 (0.842‒2.923)

Univariate logistic regression.

HR, Hazard Ratios; CI, Confidence Intervals; BCC, Basal Cell Carcınoma.

Statistically significant p values (p < 0.05) are shown in bold.

a Patients with organ transplantation or HIV (human immunodeficiency virus) infection/AIDS or hematological malignancies or those for whom immunosuppression was indicated in the medical record were considered “immunosuppressive”.

b Adapted from National Comprehensive Cancer Network guideline.^6^

### Metastasis

Tumor status evaluation revealed that 28 (3.6%) cases had RM and 4 cases (0.5%) had DM. DM was found in the liver in one patient, in the lung in one patient, and in the dura mater in two patients. The clinical and histopathological features and treatment protocols of patients who underwent RM are shown in Table 3. The most frequent RM was observed in BCCs in the periocular (20%) and auricular (16.7%) regions. RM was not observed in tumors in the L zone. The most common histopathological subtypes causing RM were basosquamous (10 cases, 33.3%), mixed (8 cases, 26.7%), and infiltrating (8 cases, 26.7%). The most common RM was located in the lymph node 15 (53.6) and bone (14 cases, 46.7%). All patients received standard surgical treatment, and most (14 cases, 71.4%) had their treatment supplemented by radiotherapy. It was noted that RM and DM occurred concurrently in all of them.

**Table 3:** Characteristics of regional metastatic basal cell carcinoma cases.

	Regional metastasis
	n = 28 (%)
**Gender**	
Female	11 (39.3)
Male	17 (60.7)
**Age at onset of primary tumor, median (range)**	79 (48‒99)
**Tumor site**	
Periocular	6 (21.4)
Auricular	5 (17.9)
Nasal	4 (14.3)
Lips	4 (14.3)
Nasolabial fold	3 (10.7)
Postauricular	2 (7.1)
Frontal, parietal, temporal, occipital	1 (3.6)
Cheeks	1 (3.6)
Neck	1 (3.6)
Chin	1 (3.6)
**Tumor location in H/M/L zone of the face**^a^	
Tumor location in H zone of the face	25 (89.3)
Tumor location in M zone	3 (10.7)
**Size of primary (mm), median (range)**	20 (4‒95)
**Invasion of primary (mm), median (range)**	12 (7‒30)
**Histopathologic features of primary**	
Basosquamous	10 (35.7)
Mixed	8 (28.6)
Infiltravite	8 (28.6)
Sclerosing/morphoeic	2 (7.1)
**Metastatic sites**	
Lymph node	15 (53.6)
Ipsilateral bone	14 (46.7)
Ipsilateral muscle	9 (30)
Soft tissues	9 (30)
Ipsilateral cartılage	7 (23.3)
Parotid gland	3 (10)
**Treatment**	
Surgical treatment	28 (100)
Radiotherapy	20 (71.4)
Radiotherapy + Chemotherapy	2 (6.6)

a H zone; “mask areas” of the face (central face, eyelids, eyebrows, periorbital region, nose, lips [skin and vermilion], chin, mandible, preauricular and postauricular skin/sulci, temple, ear), genitalia, hands, and feet. M zone; cheeks, forehead, scalp, neck, and pretibia. L zone; trunk and extremities (excluding pretibia, hands, feet, nail units, and ankles).

### Treatment

The treatments applied were as follows: Standard excision in 768 (99.9%) cases, radiotherapy in 24 (3.1%) cases, radiotherapy and chemotherapy in 4 (0.5%) cases, and topical imiquimod in only 1 (0.1%) case. Positive deep surgical margins averaged 2.53 ± 2.25 mm and positive lateral surgical margins averaged 3.19 ± 2.65 mm. The results of our proposed algorithm for evaluating the adequacy of deep and lateral surgical margins after standard excision using NCCN Guideline 2022 are summarized in Table 4. Tumors with nonaggressive histopathological subtypes that were in the H or M zone were most adequately excised (457 cases, 68%). Re-excision was performed in 127 (18.9%) patients in whom standard excision did not achieve an adequate surgical margin. When the patients in whom re-excision was performed were evaluated using our proposed algorithm, 83 (65.4%) patients had a nonaggressive histopathological subtype, were in a high-risk region in terms of tumor location, and tumor size did not differ (Table 5). Incomplete excision was present in 17 (2.2%) of the patients who underwent re-excision.

**Table 4:** Distribution of adequacy of surgical margins.^a^, ^b^

Risk factors	Surgical margin^c^	n (%)
Nonaggressive + L zone^d^ + <20 mm^e^	Excision with 4 mm margins	26 (3.9)
Nonaggressive + L zone^d^ + ≥20 mm^e^	Excision with 5 mm margins	‒
Nonaggressive + H/M zone^d^ + independent of tumor size	Excision with 5 mm margins	457 (68.0)
Aggressive + L zone^d^ + <20 mm^e^	Excision with 5 mm margins	12 (1.8)
Aggressive + L zone^d^ + ≥20 mm^e^	Surgical margin excision exceeding 5 mm	‒
Aggressive + H/M zone^d^ + independent of tumor size	Surgical margin excision exceeding 5 mm	177 (26.3)

a Recurrences and metastatic tumors were not included in this analysis.

b Fig. 1 was used when evaluating the adequacy of surgical margins.

c Surgical margins were evaluated as deep and lateral margins. Failure of any of these surgical margins was evaluated as inadequate surgical margin excision.

d H zone; “mask areas” of the face (central face, eyelids, eyebrows, periorbital region, nose, lips [skin and vermilion], chin, mandible, preauricular and postauricular skin/sulci, temple, ear), genitalia, hands, and feet. M zone; cheeks, forehead, scalp, neck, and pretibia. L zone; trunk and extremities (excluding pretibia, hands, feet, nail units, and ankles).

e Tumor size.

**Table 5:** Distribution of re-excision rates by risk groups.^a^

Risk factors	Re-excision, n (%)
Nonaggressive + L zone^b^ + <20 mm^c^	‒
Nonaggressive + L zone^b^ + ≥20 mm^c^	‒
Nonaggressive + H/M zone^b^ + independent of tumor size	83 (65.4)
Aggressive + L zone^b^ + <20 mm^c^	1 (0.8)
Aggressive + L zone^b^ + ≥20 mm^c^	‒
Aggressive + H/M zone^b^ + independent of tumor size	43 (33.9)

a Recurrences and metastatic tumors were not included in this analysis.

b H zone; “mask areas” of the face (central face, eyelids, eyebrows, periorbital region, nose, lips [skin and vermilion], chin, mandible, preauricular and postauricular skin/sulci, temple, ear), genitalia, hands, and feet. M zone; cheeks, forehead, scalp, neck, and pretibia. L zone; trunk and extremities (excluding pretibia, hands, feet, nail units, and ankles).

c Tumor size.

When evaluating the adequacy of deep and lateral surgical margins after standard excision using the 2016 NCCN guideline, tumors with the best nonaggressive histopathological subtype that was in the H or M zone and had a tumor size of less than 20 mm were found to be adequately excised (287 cases, 42.7%) (Supplementary Table 2). In 50 (39.4%) patients who underwent re-excision, the tumor had a nonaggressive histopathological subtype, was located in a high-risk region in terms of tumor location, and had a size of less than 20 mm (Supplementary Table 3).

## Discussion

Both biological and behavioral factors have been proposed to explain age dependence and gender differences in cancer incidence.^14^ In BCC, gender significantly alters age-specific BCC risk in a binary fashion: toward the end of puberty, females appear to be at a higher risk of developing BCC than males.^15^ Although BCC is more common in males than females (2:1),^1^ one study found that the risk of being diagnosed with BCC at any age increased significantly faster, by about 20%, in males than in females when gender, a constant and age-independent factor, was taken into account.^15^ In our study, the ratio between males and females was 1.4:1. Although the mean age in our study was high, the male-to-female ratio was low, which could be related to the greater likelihood of young women using tanning beds, taking care of their skin, and visiting a physician more often.^15^ However, further studies in this regard are needed because BCC is a multifactorial disease.

Since the face is constantly exposed to UV radiation, it is prone to developing skin cancer.^16^ Kang et al. reported that 93.2% of BCCs were located in the head and neck region and most commonly on the nasal unit (44 cases, 31.9%),^16^ which is similar to the results of our study. Notably, BCCs cause high morbidity and have a substantial economic impact because they occur on visible body sites.

Actinic damage (e.g., solar lentigines, actinic keratoses) caused by the long-term side effects of UV light are predictive risk factors for BCC.^17^ The authors detected actinic keratoses in 13% of our patients. One study reported that 36% of primary BCC patients had previously been diagnosed with actinic keratosis.^18^ Importantly, non-melanocytic skin cancers, actinic keratosis, and photoaging are components of a chronic process and can occur together.^19^

Beyond UV light exposure, immunosuppression (i.e., organ transplantation, HIV positivity, and hematologic malignancy) pave the way for BCC development.^20^ The incidence of BCC in transplant patients increases 5- to 10-fold due to immunosuppression.^21^ In our study, the rate of BCC development in immunosuppressed patients was 5.2%. Since our study did not include a control group, meaningful data comparisons were not possible.

Kaur et al. hypothesized that BCC occurs on a histological continuum and can be represented in a multistep model ranging from superficial to nodular to infiltrative.^22^ This finding is supported by the increased incidence of only the superficial BCC subtype at a young age and the observed mean ages by histological subtype, namely, 65 years for superficial BCC, 68 years for nodular BCC, and 71 years for infiltrative BCC.^23^ In our study, the mean age of the patients diagnosed with superficial BCC was 65 years, 68 years for nodular BCC, and 70 years for infiltrative BCC. These data thus support the theory of Kaur et al.^22^

As the most common subtype, nodular BCC accounts for approximately 50%–80% of lesions.^21^ Although superficial BCC has been confirmed as the second most common subtype,^21^ infiltrative BCC has been found to be the second most common subtype in some studies, including ours.^23^ The authors attribute this to the fact that the time since diagnosis was more than 3 years in most of our patients and that superficial and nodular BCC can transform into infiltrating BCC with advancing age.

Basosquamous cancer is rare and occurs in 1.2%–2.7% of all nonmelanoma skin cancers.^24^ In our study, this rate was 6.8%. Studies on the development of basosquamous cancer have indicated that aggressive BCCs have the potential to differentiate into basosquamous cancer over time.^25^ The authors hypothesize that the rate of basosquamous cancer in the present study was higher than that in other studies because the diagnosis time was longer in most patients.

The most significant problem for BCC patients during follow-up is tumor recurrence. Recurrence rates can range from 4% to 16.6%,^26^ and the rate in the present study was 3.9%. In another study, Luz et al. evaluated the recurrence rates after surgical treatment using the algorithm they had developed and found a recurrence rate of 1.3% for primary tumors and 1.63% for recurrent tumors during a mean follow-up period of 4.37 years.^27^ The fact that our proposed algorithm was used to evaluate retrospective data made it difficult to comment on the recurrence rates. However, the fact that the recurrence rates in both our and Luz et al.’s studies were found to be lower compared to those in the literature indicates the usability of our proposed algorithm.^12^, ^28^, ^29^

There are established risk factors for recurrence.^6^ In addition to these factors, our study found that the recurrence rate was higher in older patients. In a study by Bourlidou et al., age was not identified as a risk factor for local recurrence^28^; however, Kumar et al. concluded that patients who underwent incomplete excision were of an older age.^30^ Although age is a risk factor for BCC development, the relationship between recurrence and age has not been fully elucidated.^31^ When evaluating whether the development of ulceration in the tumor lesion is a risk factor for recurrence, no statistical difference was found in either our study or that by Lara et al.^29^ Meanwhile, using a multivariate analysis, Filho et al. showed that ulceration was correlated with tumor size.^32^ Although clinical features may change due to the slow progression of BCC and delays in consulting a physician, further studies are needed to evaluate ulceration as a risk factor.

Metastatic BCC occurs in 0.0028%–0.55% of all cases. Muscles, bones, lungs, and lymph nodes are primarily affected.^2^ In our study, the most common RM was located in the lymph nodes and bone. McCusker et al. found that RM was most common in the lymph nodes in their study and DM most common in the lungs. They further noted that the BCC patients with DM tended to be younger than those who had RM only.^9^ In our study, the median age at primary tumor onset was 79 years. Since all metastases were detected simultaneously with the primary tumor, it can be concluded that RM is detected at an older age.

The Tumor-Node-Metastasis (TNM) staging system classification, which was developed to stage BCC metastases, demonstrates the risk of metastasis with increasing tumor diameter.^33^ This risk is known to be high in giant BCC.^34^ In our study, metastases were found in all the patients with giant BCC, which is consistent with the literature. One of the risk factors for metastatic BCC is the histopathological subtype. Basosquamous BCC is an aggressive subtype and has a significant risk of metastasis (estimated at 5%).^21^ In our study, the most common metastatic subtype was basosquamous BCC. The nasal cheek, paranasal and retroauricular folds, and inner canthus are considered critical sites for BCC metastasis.^35^ The authors found the most frequent metastasis in the periocular and auricular locations. The authors believe that tumor diameter, histopathological subtype, and tumor location are critical features of metastatic BCC.

BCC located in the H and M zones is considered high-risk regardless of the tumor size. Because of the anatomic and functional limitations of these locations, narrow excision margins increase the risk of tumor recurrence. For optimal tumor clearance and maximum tissue preservation, Mohs Micrographic Surgery (MMS) or peripheral and deep-en-face methods of tumor margin assessment are recommended.^6^ In a review that evaluated the five-year cure rates between wide local excision and MMS in primary and recurrent BCC, it was reported that MMS provided a 99% cure in primary tumors and a 90%–93% cure in recurrent tumors. When wide local excision was performed, these rates were 96%–87% and 83% in primary tumors and recurrent tumors, respectively.^36^ In a study conducted by Dika et al., the recurrence rates for BCC located in the head and neck region were determined as 3.1% with MMS and 14% with conventional surgery. The researchers emphasized that, in their study, MMS was significantly superior to conventional surgery in the treatment of recurrent BCC.^37^ MMS is both an effective and cost-effective treatment option for patients with high-risk BCC.^38^ However, MMS is not readily available in many healthcare facilities worldwide. Algorithms for this treatment protocol are needed because standard surgical treatment is more accessible.

When the authors evaluated the adequacy of our patients’ surgical margins using our proposed algorithm, we observed that tumors in the high-risk regions had a higher rate of adequate surgical margins. In their study of factors influencing complete and incomplete excision, Codazzi et al. found that compared to complete excision, rates of incomplete excision were significantly higher in older patients, facial tumors, and aggressive histopathological subtypes such as sclerosing BCC and basosquamous carcinoma.^39^ Kim et al. reported that a 2–3 mm surgical margin was sufficient and safe for primary facial BCCs; however, other risk factors were not investigated in their study.^40^ The guidelines published in 2022 primarily address tumors in high-risk regions for recurrence. Notwithstanding, because of the wide variability in clinical features that may define a high-risk tumor, they emphasize that it is not possible to recommend a defined clinical cutoff for the standard excision of high-risk tumors.^6^ The data in our study provided a similar conclusion. However, because our study was performed using retrospective data, the adequacy of the surgical margins with long-term follow-up could not be evaluated.

Another treatment option for BCC is standard re-excision in patients with positive surgical margins after primary treatment or postoperative pathological evaluation. This approach is recommended for both low- and high-risk tumors.^6^ The recurrence rate following incomplete excision is 4.5 times higher than that of adequate excision,^34^ while the rate of re-excision after standard excision ranges from 9%–75%.^41^ The reasons for these different re-excision rates could not be determined from the published data because the case selection and decision-making processes for re-excision were unclear.^42^ Masud et al. reported that most of the re-excised tumors in their study were located in the facial region (most commonly on the nose and ear) and had an infiltrative histopathological subtype. However, the authors considered incomplete excision if the reports stated that the tumor extended to the margins, that is, < 0.5 mm with a focal extension, or was likely to recur.^41^ The acceptance of 0.5 mm tumor margins as adequate surgical margins in Masud et al.’s study made it difficult to interpret the data. The margins the authors used in our algorithm are more acceptable (i.e., surgical margin excision exceeding 5 mm). Robinson et al. emphasized that the decision to re-excise should take into account not only the patient factors, but also the anatomic factors (such as a high-risk region in the midface), the histopathological subtype of the tumor, and factors related to the surgical margin.^43^ Luz et al., on the other hand, reported that, in accordance with their proposed algorithm, they re-excised 6 of 7 patients with BCC who had developed recurrence, and none of them developed recurrence.^27^ In our study, the re-excision rates ranged from 0.8% to 65.4%. As shown in Table 5, regardless of the tumor size and histopathological subtype, most of the tumors that were in the high-risk region were re-excised. The factors affecting re-excision may be better elucidated through the use of our proposed algorithm in prospective studies. In the study of Luz et al., in addition to standard surgical treatment, MMS or excision with intraoperative histological analysis of the margins was preferred in appropriate cases. In our study, the re-excision rates may have been higher because only standard surgical treatment was applied.^27^

BCCs re-excised after incomplete excision account for nearly 6.0% of re-excisions.^35^ In the present study, the rate of incomplete excision after re-excision was lower, which may relate to the fact that 656 patients (85.3%) were operated on by a plastic surgeon, and 61 patients (7.9%) were operated on by a dermatologist.^44^

The guidelines published by the NCCN in 2016 were based on an examination of the adequacy of surgical margins and re-excision rates using high- and low-risk assessments that prioritized the tumor location and size characteristics. The findings showed that the optimal surgical margin was achieved at a higher rate for tumors smaller than 20 mm.^13^ Quazi et al. stated that a primary BCC of less than 20 mm with an excision margin of 4 mm appears adequate to eradicate primary BCC lesions. The authors noted that histopathological subtype, tumor location, and prior treatment should be considered.^45^ Based on these findings, it can be assumed that raising public awareness of BCC and increasing the rate of early patient registration are important ways to achieve optimal treatment and prevent a recurrence. Long-term studies using the newly published guidelines will reveal which factors are important in achieving optimal surgical margins.

The tumor diameters of the BCCs in the present study were smaller than 20 mm in most of the patients, which is consistent with other studies.^6^, ^45^ However, the diagnosis period in 61.2% of the patients exceeded three years, which again demonstrates that BCC is a slow-progressing tumor. Kricker et al. found that tumor size increased progressively in patients with BCC after first being noticed, and they emphasized the importance of early treatment.^46^

The depth of the tumor invasion is generally ignored in risk assessments for BCC recurrence.^6^ However, tumors of the aggressive subtype are known to be more invasive and resistant to treatment. In a study by Wetzel et al., the depth of invasion was statistically significantly higher in the aggressive subtypes than in the nonaggressive subtypes.^47^ In BCC, the depth of invasion should be considered when selecting a treatment option such as topical imiquimod and radiotherapy in addition to the surgical treatment option.^6^, ^47^ Based on these studies, the authors wish to highlight that the depth of BCC invasion is important for proper treatment selection and the achievement of optimal outcomes.

The present study had some limitations. First. it was a single-center, retrospective study. Second, the authors did not evaluate the recurrence and metastatic features of BCC in newly diagnosed patients. Third, the authors did not evaluate the clinical (e.g., skin type, sun protection habits, exposure to chemicals) and histopathological (e.g., perineural invasion) risk factors due to a lack of relevant data. Finally, the authors applied the developed algorithm retrospectively.

## Conclusion

The analysis of patient data showed that BCC was more common in older males, the nasal and periorbital locations were most commonly affected, and nodular and infiltrative BCC were more common with older age. In our examination of the risk factors for recurrence in patients with recurrent BCC, the authors found older age to be important. Tumor diameter, histopathological subtype, and tumor site are the defining features of metastatic BCC. When the proposed algorithm for evaluating adequate surgical margins and re-excision rates was evaluated using the 2022 NCCN data, optimal surgical outcomes were realized at a higher rate for tumors in the H and M zones. When the proposed algorithm for evaluating adequate surgical margins and re-excision rates was evaluated using the 2016 NCCN data, optimal surgical outcomes were achieved at a higher rate for tumors less than 20 mm. The authors believe that, in primary surgical treatment, the important risk factors affecting treatment success are tumor location in a high-risk region and the features of small tumors.

## Financial support

None declared.

## Authors' contributions

Yıldız Gürsel Ürün: Approval of the final version of the manuscript; study conception, design, and planning; collection, analysis, and data interpretation; writing; critical literature review and critical review of the manuscript.

Nuray Can: Approval of the final version of the manuscript; analysis, and data interpretation; writing; critical literature review.

Merve Bağış: Approval of the final version of the manuscript; analysis, and data interpretation; critical literature review.

Sezgi Sarıkaya Solak: Approval of the final version of the manuscript; data interpretation; critical review of the manuscript.

Mustafa Ürün: Approval of the final version of the manuscript; data interpretation; critical review of the manuscript.

## Conflicts of interest

None declared.

## References

[1] Basset-Seguin N., Herms F. (2020). Update in the management of basal cell carcinoma. Acta Derm Venereol.

[2] Lang B.M., Balermpas P., Bauer A., Blum A., Brölsch G.F., Dirschka T. (2019). S2k guidelines for cutaneous basal cell carcinoma — part 1: epidemiology, genetics and diagnosis. J Dtsch Dermatol Ges.

[3] Elder D.E., Massi D., Scolyer R.A., Willemze R. (2018).

[4] Fidelis M.C., Stelini R.F., Staffa L.P., Moraes A.M., Magalhães R.F. (2021). Basal cell carcinoma with compromised margins: retrospective study of management, evolution, and prognosis. An Bras Dermatol.

[5] Chren M.M., Linos E., Torres J.S., Stuart S.E., Parvataneni R., Boscardin W.J. (2013). Tumor recurrence 5 years after treatment of cutaneous basal cell carcinoma and squamous cell carcinoma. J Invest Dermatol.

[6] National Comprehensive Cancer Network [Internet] (2022). https://www.nccn.org/professionals/physician_gls/pdf/nmsc.pdf.

[7] Kimyai-Asadi A., Alam M., Goldberg L.H., Peterson S.R., Silapunt S., Jih M.H. (2005). Efficacy of narrow-margin excision of well-demarcated primary facial basal cell carcinomas. J Am Acad Dermatol.

[8] Li C.L., Chen Y.C., Yang K.C., Chen L.W. (2020). Different histopathologic profiles and outcomes between sun-exposed BCC and non-sun-exposed BCC. Sci Rep.

[9] McCusker M., Basset-Seguin N., Dummer R., Lewis K., Schadendorf D., Sekulic A. (2014). Metastatic basal cell carcinoma: prognosis dependent on anatomic site and spread of disease. Eur J Cancer.

[10] Di Lernia V., Ricci C., Zalaudek I., Argenziano G. (2013). Metastasizing basal cell carcinoma. Cutis.

[11] Ghanadan A., Abbasi A., Rabet M., Abdollahi P., Abbasi M. (2014). Characteristics of mixed type basal cell carcinoma in comparison to other BCC subtypes. Indian J Dermatol.

[12] Luz F.B., Ferron C., Cardoso G.P. (2015). Surgical treatment of basal cell carcinoma: an algorithm based on the literature. An Bras Dermatol.

[13] Bichakjian C.K., Olencki T., Aasi S.Z., Alam M., Andersen J.S., Berg D. (2016). Basal cell skin cancer, version 1.2016, NCCN clinical practice guidelines in oncology. J Natl Compr Canc Netw.

[14] Cook M.B., Dawsey S.M., Freedman N.D., Inskip P.D., Wichner S.M., Quraishi S.M. (2009). Sex disparities in cancer incidence by period and age. Cancer Epidemiol Biomarkers Prev.

[15] Bassukas I.D., Tatsioni A. (2019). Male sex is an inherent risk factor for basal cell carcinoma. J Skin Cancer.

[16] Kang K.W., Lee D.L., Shin H.K., Jung G.Y., Lee J.H., Jeon M.S. (2016). A retrospective clinical view of basal cell carcinoma and squamous cell carcinoma in the head and neck region: a single institution’s experience of 247 cases over 19 years. Arch Craniofac Surg.

[17] Verkouteren J.A.C., Ramdas K.H.R., Wakkee M., Nijsten T. (2017). Epidemiology of basal cell carcinoma: scholarly review. Br J Dermatol.

[18] Criscione V.D., Weinstock M.A., Naylor M.F., Luque C., Eide M.J., Bingham S.F. (2009). Actinic keratoses: natural history and risk of malignant transformation in the Veterans Affairs Topical Tretinoin Chemoprevention Trial. Cancer.

[19] Schmitt A.R., Bordeaux J.S. (2013). Actinic neoplasia syndrome and an update on the epidemiology of basal cell carcinoma, squamous cell carcinoma, and actinic keratosis. Curr Derm Rep.

[20] Nasr I., McGrath E.J., Harwood C.A., Botting J., Buckley P., Budny P.G. (2021). British Association of Dermatologists guidelines for the management of adults with basal cell carcinoma 2021. Br J Dermatol.

[21] Cameron M.C., Lee E., Hibler B.P., Barker C.A., Mori S., Cordova M. (2019). Basal cell carcinoma: epidemiology; pathophysiology; clinical and histological subtypes; and disease associations. J Am Acad Dermatol.

[22] Kaur P., Mulvaney M., Carlson J.A. (2006). Basal cell carcinoma progression correlates with host immune response and stromal alterations: a histologic analysis. Am J Dermatopathol.

[23] Arits A.H., Schlangen M.H., Nelemans P.J., Kelleners-Smeets N.W. (2011). Trends in the incidence of basal cell carcinoma by histopathological subtype. J Eur Acad Dermatol Venereol.

[24] Gualdi G., Soglia S., Fusano M., Monari P., Giuliani F., Porreca A. (2021). Characterization of basosquamous cell carcinoma: a distinct type of keratinizing tumour. Acta Derm Venereol.

[25] Tan C.Z., Rieger K.E., Sarin K.Y. (2017). Basosquamous carcinoma: controversy, advances, and future directions. Dermatol Surg.

[26] Kondo R.N., Gon A.D.S., Pontello Junior R. (2019). Recurrence rate of basal cell carcinoma in patients submitted to skin flaps or grafts. An Bras Dermatol.

[27] Luz F.B., Ferron C., Cardoso G.P. (2016). Analysis of effectiveness of a surgical treatment algorithm for basal cell carcinoma. An Bras Dermatol.

[28] Bourlidou E., Vahtsevanos K., Kyrgidis A., Tilaveridis I., Patsatsi A., Andreadis D. (2019). Risk factors for local recurrence of basal cell carcinoma and cutaneous squamous cell carcinoma of the middle third of the face: a 15-year retrospective analysis based on a single centre. Eur J Dermatol.

[29] Lara F., Santamaría J.R., Garbers L.E. (2017). Recurrence rate of basal cell carcinoma with positive histopathological margins and related risk factors. An Bras Dermatol.

[30] Kumar P., Orton C.I., McWilliam L.J., Watson S. (2000). Incidence of incomplete excision in surgically treated basal cell carcinoma: a retrospective clinical audit. Br J Plast Surg.

[31] Fontanillas P., Alipanahi B., Furlotte N.A., Johnson M., Wilson C.H., Pitts S.J. (2021). Disease risk scores for skin cancers. Nat Commun.

[32] Bueno Filho R., Fantini B.C., Santos C.A., Melo R.V.G., Rosan I., Chahud F. (2020). Attributes and risk factors of positive margins on 864 excisions of basal cell carcinomas: a single-center retrospective study. J Dermatolog Treat.

[33] Morgan F.C., Ruiz E.S., Karia P.S., Besaw R.J., Neel V.A., Schmults C.D. (2021). Brigham and Women’s Hospital tumor classification system for basal cell carcinoma identifies patients with risk of metastasis and death. J Am Acad Dermatol.

[34] Lackey P.L., Sargent L.A., Wong L., Brzezienski M., Kennedy J.W. (2007). Giant basal cell carcinoma surgical management and reconstructive challenges. Ann Plast Surg.

[35] Mehta K.S., Mahajan V.K., Chauhan P.S., Sharma A.L., Sharma V., Abhinav C. (2012). Metastatic basal cell carcinoma: a biological continuum of basal cell carcinoma?. Case Rep Dermatol Med.

[36] Tolkachjov S.N., Brodland D.G., Coldiron B.M., Fazio M.J., Hruza G.J., Roenigk R.K. (2017). Understanding Mohs micrographic surgery: a review and practical guide for the nondermatologist. Mayo Clin Proc.

[37] Dika E., Veronesi G., Patrizi A., De Salvo S., Misciali C., Baraldi C. (2020). It’s time for Mohs: micrographic surgery for the treatment of high-risk basal cell carcinomas of the head and neck regions. Dermatol Ther.

[38] Bittner G.C., Cerci F.B., Kubo E.M., Tolkachjov S.N. (2021). Mohs micrographic surgery: a review of indications, technique, outcomes, and considerations. An Bras Dermatol.

[39] Codazzi D., Van Der Velden J., Carminati M., Bruschi S., Bocchiotti M.A., Di Serio C. (2014). Positive compared with negative margins in a single-centre retrospective study on 3957 consecutive excisions of basal cell carcinomas. Associated risk factors and preferred surgical management. J Plast Surg Hand Surg.

[40] Kim E.S., Yang C.E., Chung Y.K. (2021). Does reduction of the oncologic safety margin for facial basal cell carcinoma result in higher recurrence rates?. Arch Craniofac Surg.

[41] Masud D., Moustaki M., Staruch R., Dheansa B. (2016). Basal cell carcinomata: risk factors for incomplete excision and results of re-excision. J Plast Reconstr Aesthet Surg.

[42] Malik V., Goh K.S., Leong S., Tan A., Downey D., O’Donovan D. (2010). Risk and outcome analysis of 1832 consecutively excised basal cell carcinoma’s in a tertiary referral plastic surgery unit. J Plast Reconstr Aesthet Surg.

[43] Robinson A.J., Walsh M., Hill C. (2016). Re: Basal cell carcinomata: risk factors for incomplete excision and results of re-excision. J Plast Reconstr Aesthet Surg.

[44] Bassas P., Hilari H., Bodet D., Serra M., Kennedy F.E., García-Patos V. (2013). Evaluation of surgical margins in Basal cell carcinoma by surgical specialty. Actas Dermosifiliogr.

[45] Quazi S.J., Aslam N., Saleem H., Rahman J., Khan S. (2020). Surgical margin of excision in basal cell carcinoma: a systematic review of literature. Cureus.

[46] Kricker A., Armstrong B., Hansen V., Watson A., Singh-Khaira G., Lecathelinais C. (2014). Basal cell carcinoma and squamous cell carcinoma growth rates and determinants of size in community patients. J Am Acad Dermatol.

[47] Wetzel M., Strickley J., Haeberle M.T., Brown T.S. (2019). Depth of invasion of aggressive and nonaggressive basal cell carcinoma. J Clin Aesthet Dermatol.

