# Supplementary material for: Adequacy of surgical margins, re-excision, and evaluation of factors associated with recurrence: a retrospective study of 769 basal cell carcinomas
Source: An Bras Dermatol. 2023 Mar 16;98(4):449–59. doi: 10.1016/j.abd.2022.07.005 (PMC10334344; doi:10.1016/j.abd.2022.07.005)
Supplement: Supplementary file 1 [file mmc1.docx]

ABD-D-22-00254_Supplementary Material

**Supplement Table 1** Demographic and clinical data and tumor features of patients with basal cell carcinoma.

|  | **n=769 (%)** |
| --- | --- |
| **Patient Demographics** |  |
| Gender |  |
| Male | 455 (59.2) |
| Female | 314 (40.8) |
| Age at diagnosis, median (range) | 69 (23‒99) |
| Time since diagnosis |  |
| Under 1 year | 10 (1.3) |
| 1–3 years | 45 (5.9) |
| Over 3 years | 614 (79.8) |
| Unknown | 100 (13.0) |
| **Clinical Features** |  |
| Ulcer | 437 (56.8) |
| Actinic keratosis | 100 (13) |
| Immunosuppression^a^ | 40 (5.2) |
| **Tumor Features** |  |
| Tumor size (mm, max dimension), mean (SD) | 12.09 (±9.76) |
| Depth of invasion (mm), mean (SD) | 3.92 (±4.12) |
| Location^b^ |  |
| H zone | 554 (72.0) |
| M zone | 163 (21.2) |
| L zone | 52 (6.8) |
| Sun-exposed body site | 723 (94) |
| Sun-protective body site | 46 (6) |
| **Tumor status** |  |
| Local recurrence | 74 (9.6) |
| Regional metastasis | 28 (3.6) |
| Distant metastasis | 4 (0.5) |
| Patients with a multiple BCC |  |
| Single | 665 (86.5) |
| Multiple | 104 (13.5) |

^a^ Patients with organ transplantation or HIV (human immunodeficiency virus) infection/AIDS or hematological malignancies or those for whom immunosuppression was indicated in the medical record were considered “immunosuppressive”.

^b^ H zone; “mask areas” of the face (midface, eyelids, eyebrows, periorbital region, nose, lips [skin and vermilion], chin, mandible, preauricular and postauricular skin/sulci, temple, ear), genitalia, hands, and feet. M zone; cheeks, forehead, scalp, neck, and pretibia. L zone; trunk and extremities (excluding pretibia, hands, feet, nail units, and ankles).

**Supplement Table  2** Distribution of adequacy of surgical margins^a,b^.

| **Risk factors** | **Surgical margin^c^** | **n (%)** |
| --- | --- | --- |
| Nonaggressive^d^ + Low-risk^e^ + 20 mm^f^ < | Excision with 4 mm margins | 157 (23.4) |
| Nonaggressive^d^ + Low-risk^e^ + 20 mm^f^ ≥ | Excision with 4 mm margins | 0 (0) |
| Nonaggressive^d^ + High-risk^e^ + 20 mm^f^ < | Excision with 4 mm margins | 287 (42.7) |
| Nonaggressive^d^ + Low-risk^e^ + 20 mm^f^ ≥ | Excision with 5 mm margins | 39 (5.8) |
| Aggressive^d^ + Low-risk^e^ + 20 mm^f^ < | Excision with 5 mm margins | 55 (8.2) |
| Aggressive^d^ + Low-risk^e^ + 20 mm^f^ ≥ | Surgical margin excision exceeding 5 mm | 0 (0) |
| Aggressive^d^ + High-risk^e^ +20 mm^f^ < | Surgical margin excision exceeding 5 mm | 112 (16.7) |
| Aggressive^d^ + High-risk^e^ + 20 mm^f^ ≥ | Surgical margin excision exceeding 5 mm | 22 (3.3) |

^a^ Recurrences and metastatic tumors were not included in this analysis.

^b^ The National Comprehensive Cancer Network Guideline for Basal Cell Skin Cancer published in 2016 was used in evaluating the adequacy of surgical margins.[13]

^c^ Surgical margins were evaluated as deep and lateral margins. Failure of any of these surgical margins was evaluated as inadequate surgical margin excision.

^d^ Nonaggressive histopathological subtypes: Nodular, superficial, and other rare nodular BCC subtypes such as adenoid, keratotic, infundibulocystic, and fibroepithelioma of Pinkus. Aggressive histopathological subtypes: Infiltrative, micronodular, sclerosing/morpheic, basosquamous, or mixed.

^e^ The low-risk group includes tumors smaller than 20 mm in the L zone, < 10 mm in the M zone, and < 6 mm in the H zone. The high-risk group includes tumors ≥ 20 mm in the L zone, ≥10 mm in the M zone, and ≥ 6 mm in the H zone.

^f^ Tumor size.

**Supplement Table  3** Distribution of re-excision rates^a,b^.

| **Risk factors** | **n (%)** |
| --- | --- |
| Nonaggressive^c^ + Low-risk^d^  + 20 mm^e^ < | 29 (22.8) |
| Nonaggressive^c^ + Low-risk^d^ + 20 mm^e^ ≥ | 0 (0) |
| Nonaggressive^c^ + High-risk^d^ + 20 mm^e^ < | 50 (39.4) |
| Nonaggressive^c^ + Low-risk^d^ + 20 mm^e^ | 4 (3.1) |
| Aggressive^c^ + Low-risk^d^ + 20 mm^e^ < | 15 (11.8) |
| Aggressive^c^ + Low-risk^d^ + 20 mm^e^ ≥ | 0 (0) |
| Aggressive^c^ + High-risk^d^ + 20 mm^e^ < | 28 (22) |
| Aggressive^c^ + High-risk^d^ + 20 mm^e^ ≥ | 1 (0.8) |

^a^ Recurrences and metastatic tumors were not included in this analysis.

^b^ The National Comprehensive Cancer Network Guideline for Basal Cell Skin Cancer published in 2016 was used in evaluating the adequacy of surgical margins.[13]

^c^ Nonaggressive histopathological subtypes: Nodular, superficial, and other rare nodular BCC subtypes such as adenoid, keratotic, infundibulocystic, and fibroepithelioma of Pinkus. Aggressive histopathological subtypes: Infiltrative, micronodular, sclerosing/morpheic, basosquamous, or mixed.

^d^ The low-risk group includes tumors smaller than 20 mm in the L zone, < 10 mm in the M zone, and < 6 mm in the H zone. The high-risk group includes tumors ≥ 20 mm in the L zone, ≥ 10 mm in the M zone, and ≥ 6 mm in the H zone.

^e^ Tumor size.
